# Pre-Eclampsia-Induced Maternal Liver Dysfunction: Systematic Review, Meta-Analysis and Meta-Regression of Observation Studies

**DOI:** 10.3390/life16020223

**Published:** 2026-01-29

**Authors:** Kay-Lee E. Strauss, Wendy N. Phoswa, Kabelo Mokgalaboni

**Affiliations:** Department of Life and Consumer Sciences, College of Agriculture and Environmental Sciences, University of South Africa, Florida Campus, Calabash Building, Office 02-047, Roodepoort 1710, South Africa

**Keywords:** pre-eclampsia, liver function, aspartate aminotransferase, alanine aminotransferase, alkaline phosphatase, bilirubin, hepatic dysfunction

## Abstract

**Introduction**: Pre-eclampsia (PE) is a pregnancy-related hypertensive condition defined by the onset of hypertension after 20 weeks of gestation that is associated with proteinuria and maternal organ damage or uteroplacental dysfunction. It continues to be a leading cause of maternal and perinatal morbidity and mortality globally. PE is linked to systemic inflammation, endothelial dysfunction, and oxidative stress, which may compromise hepatic function. **Aim**: This meta-analysis assesses the impact of PE on maternal liver function by evaluating hepatic biomarkers, including aspartate aminotransferase (AST), alanine aminotransferase (ALT), alkaline phosphatase (ALP), and total serum bilirubin. **Methods**: This meta-analysis of observational studies in Epidemiology (MOOSE) involved a search of PubMed and Scopus and manual screening of studies published between 2000 and 2025. Eligible studies included cross-sectional, case–control, and cohort designs. The quality of the studies was evaluated using the Newcastle–Ottawa Scale. Statistical analysis was conducted using the online meta-analysis, Jamovi version 2.6.44, and IBM SPSS Statistics version 30, and effect estimates were reported as standardized mean differences (SMDs) with 95% confidence intervals (CIs). **Results**: Forty-five studies, comprising 257,929 women 9420 with PE; 248,509 normotensive, were included. Women with PE had elevated AST, MD = 1.81 (95% CI: 1.51 to 2.10; *p* < 0.0001) and ALT, SMD = 1.73 (95% CI: 1.38 to 2.07; *p* < 0.0001); ALP, SMD = 1.43 (95% CI: 0.97 to 1.88; *p* < 0.0001); and total serum bilirubin (TSB), SMD = 0.62 (95% CI: 0.36 to 0.88; *p* < 0.0001) in comparison to normotensive controls. In the meta-regression, maternal age and quality were significant moderators, with older age and high-quality studies associated with smaller and larger effect sizes, respectively, for ALP (*β* = −0.720 and *β* = 1.444) and TSB (*β* = −0.304 and *β* = 0.761). For every 1-unit increase in body mass index, there was a significant 0.406-unit decrease in ALT effect size. **Conclusions**: PE is significantly associated with elevated maternal hepatic enzyme levels, indicating hepatocellular damage and impaired liver function. These findings emphasise the necessity for routine liver function monitoring in pregnant women with hypertensive disorders.

## 1. Introduction

Pre-eclampsia (PE) is a hypertensive disorder occurring during pregnancy, characterised by new-onset hypertension defined as systolic blood pressure (SBP) ≥ 140 mmHg and diastolic blood pressure (DBP) ≥ 90 mmHg after 20 weeks of gestation [1,2,3]. It is accompanied by either proteinuria or maternal organ dysfunction and or uteroplacental dysfunction. It continues to be a significant contributor to maternal and perinatal morbidity and mortality globally, impacting around 2–8% of pregnancies [4]. Pregnancy is associated with substantial metabolic, haemodynamic, and hormonal changes that promote maternal well-being and foetal growth [5]. In most cases, these changes do not result in substantial impairment. However, in the case of PE, these gestational changes may be significant, resulting in compromised liver function. This is attributed to the pathophysiology of PE, which is centred around the abnormal placental growth and function, resulting in systemic inflammation, oxidative stress, and endothelial dysfunction [6,7,8]. Moreover, inflammation is associated with liver dysfunction, as the liver serves as both a major immunological organ and a target of inflammatory mechanisms [9]. The liver is an essential organ in human physiology, playing a vital role in metabolism, detoxification, protein synthesis, and the regulation of biochemical homeostasis [10,11]. The ability of the liver to maintain stable levels of circulating biomarkers, such as aspartate aminotransferase (AST), alanine aminotransferase (ALT), alkaline phosphatase (ALP), and bilirubin, is essential, as these are sensitive indicators of hepatic integrity and function [12]. Due to its vital role, any impairment in liver function can lead to systemic repercussions, especially during pregnancy, when maternal physiology experiences heightened metabolic and haemodynamic demands [13].

Initiation of an inflammatory response promotes the activation of liver macrophages (Kupffer cells) and neutrophils [14]. These cells secrete pro-inflammatory cytokines such as tumour necrosis factor-alpha (TNF-α), interleukin-6 (IL-6), and interleukin-1β (IL-1β), as well as reactive oxygen species [15]. This sequence of mediators induces hepatocellular oxidative stress and damage, which presents as elevated liver enzyme levels [16]. Therefore, a timely identification and intervention for PE are crucial to prevent complications and to preserve the health of both the mother and the infant. The hepatic symptoms of PE differ from moderate biochemical anomalies, such as increased liver enzymes, to severe complications such as hepatic rupture, infarction, and in severe cases, HELLP (Haemolysis, Elevated Liver enzymes, and Low Platelets) syndrome [17]. These complications not only compromise the mother’s health but also complicate clinical management and affect pregnancy outcomes. Moreover, liver dysfunction exacerbates negative pregnancy outcomes, such as preterm delivery, intrauterine growth restriction, and stillbirth, highlighting the combined risk to mother and foetal health. Therefore, this study aims to examine the effects of PE on maternal liver function by analysing changes in hepatic biomarkers (AST, ALT, ALP, and bilirubin).

## 2. Methodology

### 2.1. Study Design

This study is a meta-analysis of observational studies and adheres to the Meta-analysis of Observational Studies in Epidemiological (MOOSE) guideline [18] (Supplementary File S1). This study also followed the PICO framework in designing the Preferred Reporting Items for Systematic Reviews and Meta-Analysis (PRISMA) flow chart [19]. It also adheres to PECOS criteria as outlined in Table 1.

### 2.2. Search Strategy, Literature Search, and Selection Criteria

Two independent researchers (KES and KM) conducted an extensive literature search using the PubMed and Scopus databases, employing Medical Subject Headings (MeSH) terms to identify relevant published studies, as well as a comprehensive bibliography search. The MeSH terms and Boolean operators used for the search included “Liver Function” OR “Aspartate Aminotransferase” OR “AST” OR “Alanine Aminotransferase” OR “ALT” OR “Alkaline Phosphatase” OR “ALP” OR “Bilirubin” AND “Pre-eclampsia”. The review focused on studies published between 2000 and 2025 to identify evidence that reflects current diagnostic criteria, clinical practices, and methodological standards. Studies published before 2000 were excluded because of changes in the definition and management of PE, which could introduce variability.

### 2.3. Data Extraction and Quality Assurance

Two independent researchers (KES and KM) used a preformatted Excel spreadsheet to extract data from each study. Two researchers evaluated the two sheets, and in cases of disagreement over key items, a third researcher, WNP, was consulted to review the study and the disputed variables before a decision was reached. The primary data obtained from each study comprised the lead author’s family name, country of publication, study design, population size, blood pressure (SBP, DBP) and body mass index (BMI) of the participants, and the findings, mean, standard deviation (SD), and sample size of AST, ALT, ALP, and total serum bilirubin. The Newcastle–Ottawa Scale was used to assess the quality of the included case–control, cohort, and cross-sectional studies [20]. This tool focuses on selection, comparability, exposure, and outcome based on the design. Each item was rated with a shaded star or no empty star, and overall quality was judged accordingly. The study was considered high quality (low risk of bias) if it scored 8–9 stars, moderate if it scored 5–7 stars, and low if it scored 0–4 stars.

### 2.4. Statistical Analysis

For the meta-analysis, we used online meta-analysis software [21]. Jamovi version 2.6.44 and IBM SPSS Statistics version 30 were used for meta-regression and subgroup analysis, respectively. We calculated the effect estimates for all indicators by computing the mean, SD, and sample size for each study group. The mean and SD were estimated from the median and range using the protocols reported by Hozo et al. (2005) [22]. When the study reported the standard error of the mean (SEM), SD was estimated as SD = SEM × √n [23]. If the study reported the median and interquartile range (IQR), the mean was used as the median for a larger sample, and the SD was estimated as SD = IQR/1.35. In studies with multiple PE groups (moderate, mild, or severe), we used the Cochrane method to combine them into a single PE group. We employed the *I*^2^ statistic test to assess statistical heterogeneity [24,25]. *I*^2^ values of ≤50% and ≥75% were categorised as low and substantial statistical heterogeneity, respectively [26]. Moreover, the Egger regression test, Beggs and Mazumdar’s rank correlation, trim and fill, including safe-fail N assessment, were used to assess and adjust for publication bias. Sensitivity analysis was performed using one-study-exclusion methods to assess the stability of the effect size [27]. Subgroup analyses were conducted by study design, maternal age, gestational age at diagnosis of PE, study quality, BMI, and continent of publication. For meta-regression, different moderators including (maternal age and BMI, duration of gestation at time of diagnosis of PE, study design, and content of publication) were explored. A *p*-value below 0.05 was considered statistically significant.

## 3. Results

### 3.1. Literature Search and Screening

**Figure 1:**
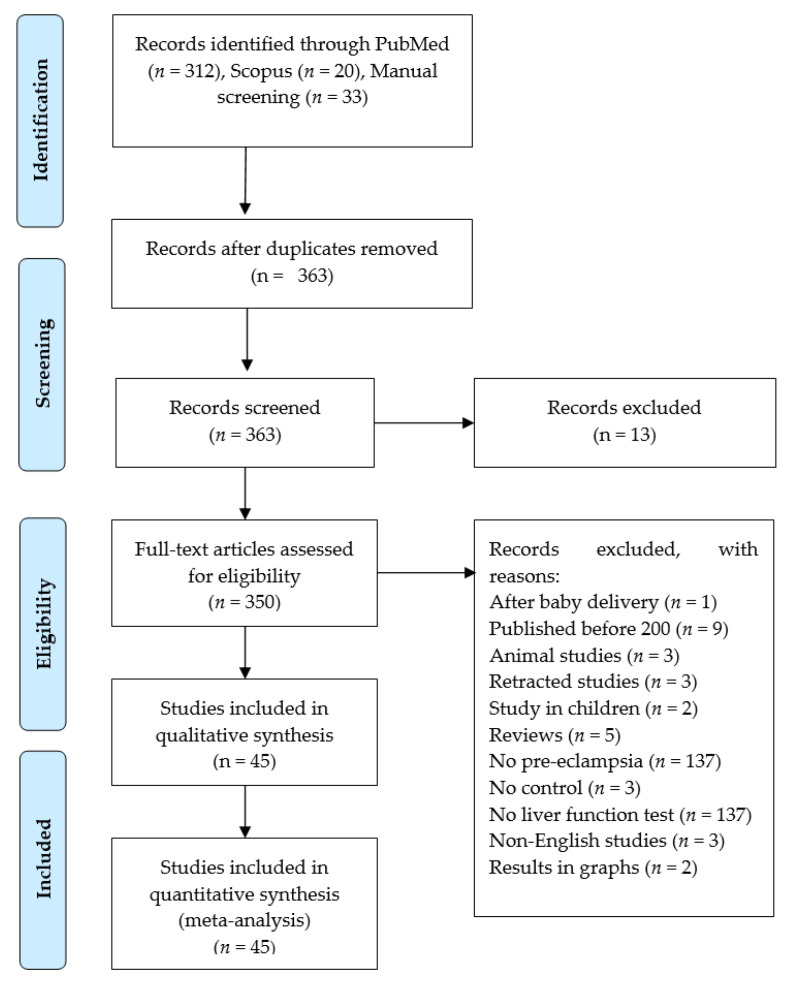
A flowchart illustrating the study selection process.

The preliminary search on the PubMed and Scopus databases produced 332 records. Furthermore, we conducted a bibliographic search and identified 33 relevant studies, bringing the total to 365 for review. Initially, we used an Excel spreadsheet to group all records; 2 duplicates were thus identified and excluded. Of the records that underwent initial full screening, 13 were excluded because their titles and abstracts were deemed irrelevant to the topic. Additionally, 305 were excluded for various reasons, including studies in animals, children, irrelevant markers reported, results presented graphically, not containing a control group, article retracted, no liver function test or pre-eclampsia present, the study was conducted after delivery of the baby, study not published in English, articles published before 2000, and review articles. Hence, 45 studies [28,29,30,31,32,33,34,35,36,37,38,39,40,41,42,43,44,45,46,47,48,49,50,51,52,53,54,55,56,57,58,59,60,61,62,63,64,65,66,67,68,69,70,71,72] were deemed relevant as they satisfied the PECOS criteria outlined in Table 1. To address any discrepancies and prevent bias, a third independent researcher (WNP) participated in the screening and selection process. Refer to Figure 1 for a comprehensive explanation of the screening and selection procedure.

### 3.2. Characteristics of the Studies Included

**Figure 2:**
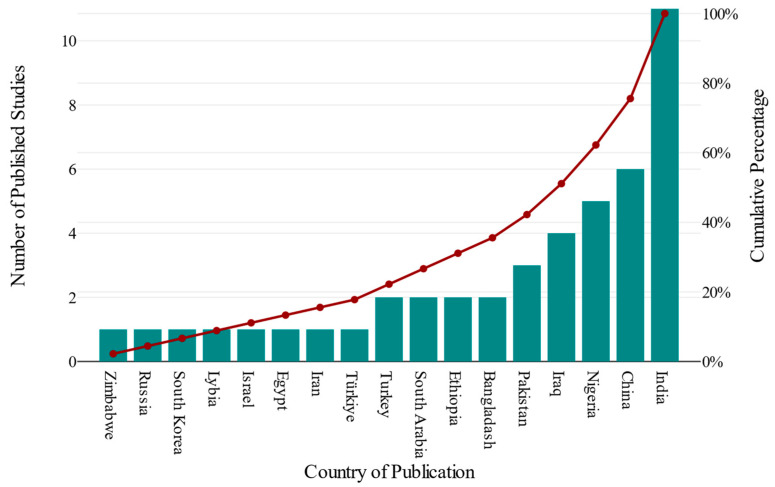
Geographical distribution of publications.

We analysed data from 45 studies [28,29,30,31,32,33,34,35,36,37,38,39,40,41,42,43,44,45,46,47,48,49,50,51,52,53,54,55,56,57,58,59,60,61,62,63,64,65,66,67,68,69,70,71,72] published in peer-reviewed journals between 2000 and 2025, evaluating the effect of PE on liver function in pregnant women. The sample sizes showed significant heterogeneity across research, ranging from small samples [51] to larger population studies [40,42,43]. The overall sample comprised 257,929 pregnant women: 9420 with PE and 248,509 normotensives. The published studies employed various designs, including 26 cross-sectional [28,29,30,31,38,39,46,49,50,51,52,53,55,56,57,58,59,60,61,62,63,64,65,66,67,68,71], 11 case–control [32,33,34,35,36,37,45,48,69,70,72], and 8 cohort designs [40,41,42,43,44,47,54,71]. Studies from 15 countries were analysed (Figure 2), with the majority from India [31,33,55,56,59,60,61,63,66,69,72], China [32,40,43,47,54,70], Nigeria [28,37,57,67,68], Iraq [34,39,58,62], Pakistan [41,50,52], Ethiopia [29,49], Bangladesh [30,35], Zimbabwe [65], Turkey [36,45], Libya [46], Iran [48], Saudi Arabia [38,64], Egypt [51], Korea [42], Israel [44], Russia [53] and Türkiye [71] (Figure 2). The average blood pressure of the PE group was 150.20 ± 15.14 mmHg systolic and 96.93 ± 9.40 mmHg diastolic. In contrast, the normotensive group had an average SBD and DBP of 115.42 ± 8.95 mmHg and 72.93 ± 6.55 mmHg, respectively. The average maternal age in the PE group reported in 33 studies was 29.65 ± 5.14 years, compared with 32 studies in normotensive women, which reported an average age of 28.75 ± 5.06 years. Moreover, the average BMI of the 17 students in the PE group was 26.77 ± 4.17 kg/m^2^, whereas in the normotensive group, it was 24.22 ± 3.64 kg/m^2^. The overall features of the included studies are presented in Table 2.

### 3.3. AST Levels in Pregnant Women with Pre-Eclampsia Versus Normotensive Pregnant Women

**Figure 3:**
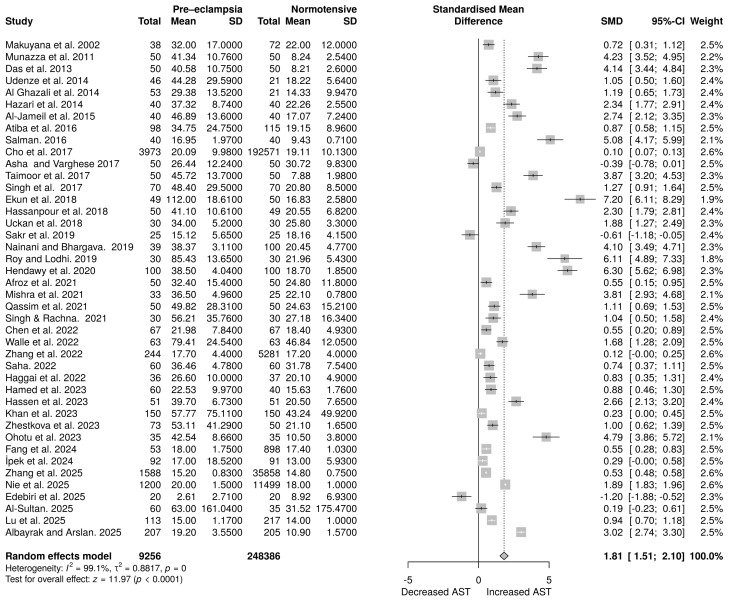
Random effects meta-analysis assessing the impact of PE on aspartate aminotransferase levels [28,29,31,32,33,34,36,37,38,39,40,42,43,44,45,46,47,48,49,50,51,52,53,54,55,56,57,58,59,60,61,62,63,64,65,66,67,68,69,70,71,72].

A total of 42 studies examined AST levels, including 9256 participants in the PE group and 248,386 subjects in the normotensive group. The analysis employed a random-effects model, revealing a statistically significant difference between the two groups (Figure 3), with an overall SMD of 1.81 (95% CI: 1.51 to 2.10). The overall effect estimates were statistically significant (*p* < 0.0001). However, significant heterogeneity was noted (*p* = 0), with an *I*^2^ = 99.1%.

### 3.4. ALT Levels in Pre-Eclampsia Compared to Normotensive

**Figure 4:**
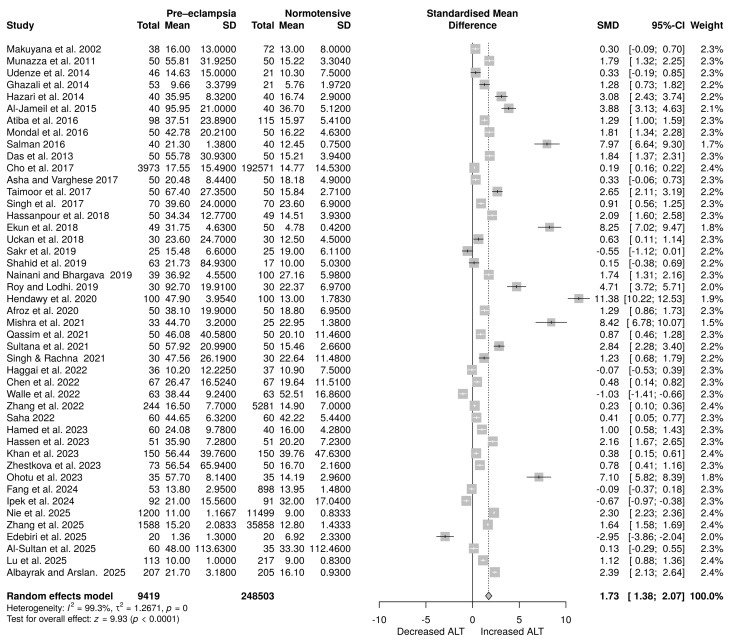
Random effects meta-analysis assessing the impact of PE on alanine aminotransferase levels [28,29,30,31,32,33,34,35,36,37,38,39,40,41,42,43,44,45,46,47,48,49,50,51,52,53,54,55,56,57,58,59,60,61,62,63,64,65,66,67,68,69,70,71,72].

A total of 45 studies examined ALT levels, comprising 9419 participants in the PE group and 248,503 participants in the normotensive group. The analysis employed a random-effects model due to high heterogeneity (*I*^2^ = 99.3%; *p* = 0.0). The results revealed a statistically significant difference between the two groups, with an SMD of 1.73 (95% CI: 1.38 to 2.07), *p* < 0.0001, as shown in Figure 4.

### 3.5. ALP Levels in Pre-Eclampsia Compared to Normotensive Pregnant Women

**Figure 5:**
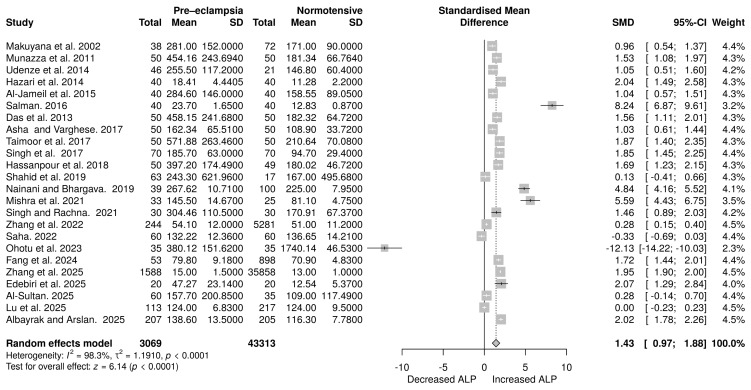
Random effects meta-analysis assessing the impact of PE on alkaline phosphatase levels [33,37,41,43,47,48,50,52,54,55,56,57,58,59,60,62,64,65,66,67,69,70,71,72].

Twenty-four studies analysed ALP levels, comprising 3069 participants in the PE group and 43,318 subjects in the normotensive group. The random effects model meta-analysis revealed an increase in ALP, with an SMD of 1.43 (95% CI: 0.97 to 1.88), as shown in Figure 5. The overall effect results demonstrated a statistically significant difference between the groups (*p* < 0.0001). However, substantial heterogeneity was observed (*p* < 0.01), with an *I*^2^ value of 98.3%.

### 3.6. Total Bilirubin Levels in Pre-Eclampsia Versus Normotensive Pregnant Women

**Figure 6:**
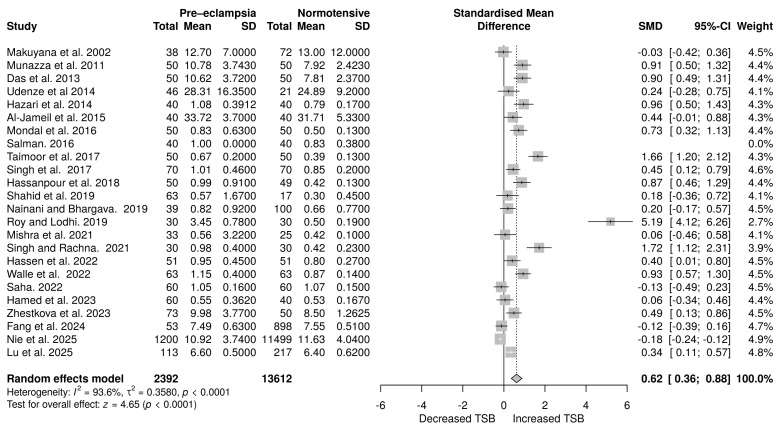
Random effects meta-analysis assessing the impact of PE on total serum bilirubin levels in pregnant women [29,30,33,37,40,41,46,47,48,49,50,52,53,55,56,58,59,60,61,64,65,66,70,72].

In total, 24 studies examined total serum bilirubin (TSB) levels, including 2392 pregnant women with PE and 13,612 normotensive women. A random-effects meta-analysis was employed, and the effect estimates revealed an increased TSB (SMD = 0.62, 95% CI: 0.36 to 0.88). The overall effect was statistically significant (*p* < 0.0001). Significant heterogeneity was observed (*p* < 0.0001), with an *I*^2^ value of 93.6% (Figure 6).

### 3.7. Publication Bias Assessments

**Figure 7:**
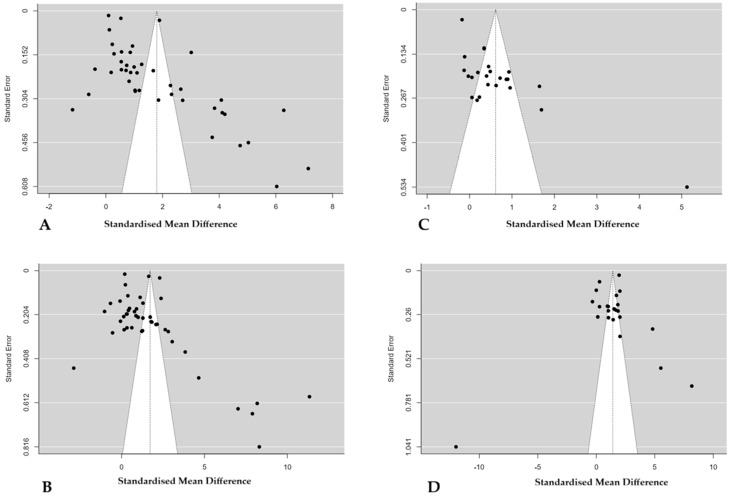
Funnel plots illustrate the possible publication bias among the studies included in the meta-analysis. (**A**) Studies that analysed AST levels in pregnant women with PE and normotensive pregnant women. (**B**) Studies that analysed total bilirubin levels in pregnant women with PE and normotensive pregnant women. (**C**) Studies that analysed ALT levels in pregnant women with PE and normotensive pregnant women. (**D**) Studies that analysed ALP levels in pregnant women with PE and normotensive pregnant women. The black dot indicates an individual study. A gray colour region indicates a pseudo-confidence interval region.

This meta-analysis included more than 10 studies, allowing for the assessment of publication bias. Funnel plot inspection, Egger’s and Beggs’ tests indicated potential publication bias for AST levels (Figure 7A) (E value = 9.596, *p* < 0.001) and Begg and Mazumdar’s rank correlation (value = 0.422, *p* < 0.001). Moreover, the trim-and-fill value was 0.00, with a fail-safe N of 42,802 and *p* < 0.001. Similarly, for ALT (Figure 7C), the Egger regression test suggested evidence of publication bias (value = 10.430, *p* < 0.001), and the Beggs’ value was 0.335 (*p* = 0.001). The Fail-Safe N value was 51,532, *p* < 0.001, whereas the trim and fill value was 0.00. Likewise, for TSB levels, funnel plot inspection (Figure 7B), Egger regression (value = 6.172, *p* < 0.001), and Beggs’s test (value = 0.297, *p* = 0.044) all indicated publication bias. Moreover, the evidence showed a significantly high fail-safe N value (215,000; *p* = 0.001) and a trim-and-fill value of 0.00. In contrast, for ALP, visual inspection of the funnel plot suggested no evidence of publication bias (Figure 7D). This is confirmed statistically by the Egger regression test (*p* = 0.05, regression coefficient = −0.2009) and the Beggs test (*p* = 0.131, test statistic = 0.225). The fail-safe N value was 3965, with a trim-and-fill of 0.000. These results altogether suggest that the pooled effect size from the ALP meta-analysis is robust and unlikely to be substantially influenced by publication bias.

### 3.8. Subgroup Analysis

A subgroup analysis was conducted to identify sources of heterogeneity in AST, ALT, ALP, and bilirubin levels by study design, maternal age, period of gestation during diagnosis of PE, continent, study quality, and BMI, as presented in Supplementary File S2, Table S1. For the AST subgroup, factors including study design, quality, continent, BMI, gestational duration, and maternal age did not account for the observed heterogeneity, as they did not reduce heterogeneity (Supplementary File S2, Table S1). Similarly, for ALT and ALP, none of these factors altered heterogeneity. In contrast, in the TSB subgroup, we found that study design, particularly cohort (*I*^2^ = 0%) and gestational age at diagnosis (*I*^2^ = 51.1%), were potential sources of observed heterogeneity.

### 3.9. Meta-Regression

The meta-regression output is presented in Table 3. For AST, among these moderators, study design (*β* = −0.630, *p* < 0.001) was the significant moderator. Additionally, the significant positive regression coefficient (*β* = 1.084, *p* < 0.001) indicates that for every 1-unit increase in the quality score, the effect size increases by 0.761 on average (Table 3). Therefore, there was no evidence that maternal age, gestational age at diagnosis, continent, or BMI explained the heterogeneity observed for AST. Similarly, we found that study design (*β* = 1.084, *p* < 0.019) and quality (*β* = 0.729, *p* = 0.039) were significant moderators of the ALT effect size. Moreover, the significant negative regression coefficient on ALT BMI (*β* = −0.406, *p* = 0.006) suggests that for every 1-unit increase in BMI, the effect size decreases by 0.406 units. For ALP, maternal age (*β* = −0.720, *p* = 0.010), quality (*β* = 1.444, *p* = 0.003), and continent (*β* = 2.07, *p* < 0.0001) were significant moderators. For TSB, study design (*β* = −0.384, *p* = 0.004), quality (*β* = 0.761, *p* < 0.001), and maternal age (*β* = −0.304, *p* = 0.019) were significant moderators.

### 3.10. The Sensitivity Analysis for Robustness and Stability of Effect Size

For AST, excluding four studies individually resulted in a change in effect size. Briefly, the exclusion of Salman [58] led to SMD = 1.74, 95% CI (1.44 to 2.03, *p* < 0.0001), Ekun [68], SMD = 1.70, 95% CI (1.41 to 1.99, *p* < 0.0001), Roy and Lodhi [61], SMD = 1.73, 95% CI (1.43 to 2.02, *p* < 0.0001) and Hendawy [38], SMD = 1.70, 95% CI (1.41 to 1.99, *p* < 0.0001). For ALT, six studies changed the effect size. The exclusion of Ohotu [57] changed effext to SMD = 1.65, *p* < 0.0001; Edebiri [67], SMD = 1.82, *p* < 0.0001; Mishra [33], SMD = 1.62, *p* < 0.0001; Hendawy [38], SMD = 1.54, *p* < 0.0001; Ekun [68], SMD = 1.060, *p* < 0.0001; and Salman [58], SMD = 1.61, *p* < 0.0001. We also noted that excluding only 4 of 25 studies for ALP changes the effect size. For instance, the effect changed for the exclusion of Salman [58] SMD = 1.21 (0.76 to 1.65, *p* < 0.0001), Mishra [33], SMD = 1.28, 95% CI (0.82 to 1.73, *p* < 0.0001); Ohotu [57], SMD = 1.73, 95% CI (1.30 to 2.17, *p* < 0.0001); Nainani [56], SMD = 1.28, 95% CI (0.83 to 1.73, *p* < 0.0001). For TSB, only excluding the Roy and Lodhi (2019) study [61] changed the effect size to SMD = 0.49 (0.26 to 0.72, *p* < 0.0001), a 21% decrease from the initial effect in the same direction.

### 3.11. Quality Assessment of Included Observational Studies

The quality of observational studies is presented in Supplementary File S2, Tables S2–S4. For the seven evaluated cohorts, six studies received 10 stars across all domains, and one study received 8 stars due to poor reporting of the adequacy of follow-up; however, all were rated as high quality (Supplementary File S2, Table S2). In 26 cross-sectional studies, 20 scored 7 stars, and one scored 5 stars [68], all of which were considered of moderate quality. Four studies received 8 stars, and 1 study scored 9 stars; thus, all were rated as high quality (Supplementary File S2, Table S3). For case–control studies, 11 studies scored 8–10 stars and were regarded as high quality, except for a study by Asha and Varghese (2017) [69], which was rated 6/10, indicating moderate quality (Supplementary File S2, Table S4). Overall, 51% of the studies were rated as high quality and 49% rated as moderate quality.

## 4. Discussion

This systematic review and meta-analysis evaluated data from 45 observational studies investigating the impact of PE on maternal liver function by analysing four biochemical markers: AST, ALT, ALP, and TSB. The results demonstrate that pregnant women with PE have elevated levels of liver function enzymes when compared to normotensive pregnancies. These results suggest that PE may impair hepatic function and induce hepatocellular stress in pregnancy. Our findings are consistent with evidence from previous studies, which show a consistent trend in the association between PE and liver dysfunction [29,31,32,40,72]. This is also confirmed by HIV patients, irrespective of treatment [73,74]. The overall data demonstrated statistically significant increases in AST, ALT, ALP, and bilirubin levels in women with PE, demonstrating hepatocellular damage and supporting the notion that systemic inflammation and endothelial dysfunction impair hepatocyte function [75].

**Figure 8:**
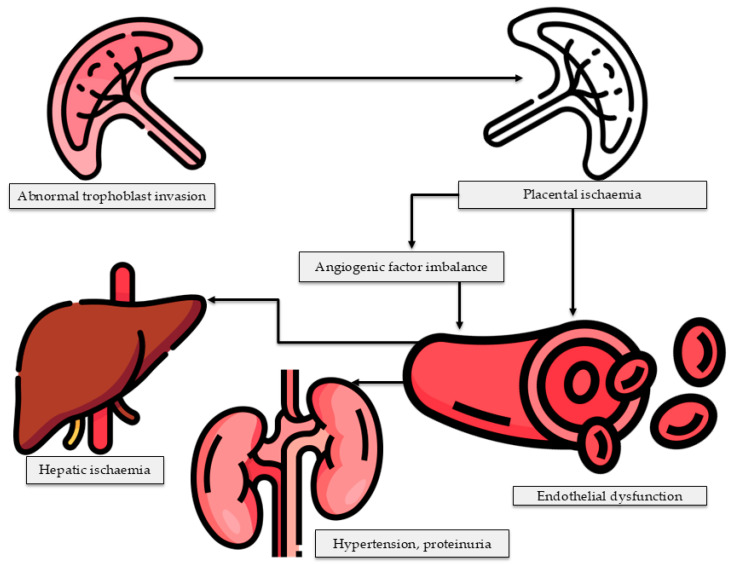
Schematic depiction of the disparity between angiogenic and anti-angiogenic factors resulting in endothelial dysfunction. This malfunction leads to multi-organ involvement in pre-eclampsia, impacting essential maternal organs such as the liver, kidney, and brain. Figure created using FLATICON and Microsoft PowerPoint.

Although the mechanism by which PE initiates liver dysfunction is complex, emerging evidence distinguishes two primary pathogenic subtypes of PE, with distinct mechanisms promoting liver dysfunction that depend on the stage of PE: early- and late-onset PE [76]. Early-onset PE is associated with defects in trophoblast invasion, resulting in abnormal placentation, placental ischaemia, and endothelial injury [76,77]. It’s essential to note that the trophoblast plays a crucial role in the attachment of the developing embryo to the endometrium, providing protection and forming part of the placenta [78]. However, any impairment in its function would disrupt all these delicate functions. On the other hand, defects in the trophoblast cause an imbalance in the production of angiogenic and antiangiogenic factors. Whereby, the release of antiangiogenic factors, such as soluble fms-like tyrosine kinase-1 receptor (sFlt-1), is increased compared to angiogenic factors such as vascular endothelial growth factor (VEGF) and placental growth factor (PGF) [79,80,81]. The imbalance between angiogenic and anti-angiogenic factors ultimately leads to endothelial dysfunction, which affects maternal organs, including the heart, liver, and kidneys, and, in severe cases, the brain (Figure 8). On the other hand, late-onset PE is associated with maternal metabolic dysfunction and systemic inflammation. Therefore, these contrasting pathways suggest that liver dysfunction in early-onset PE may arise due to placental ischemia-induced hepatic endothelial damage [74,82,83,84], whereas in late-onset PE, maternal metabolic abnormalities and chronic inflammation may exert a greater influence.

**Figure 9:**
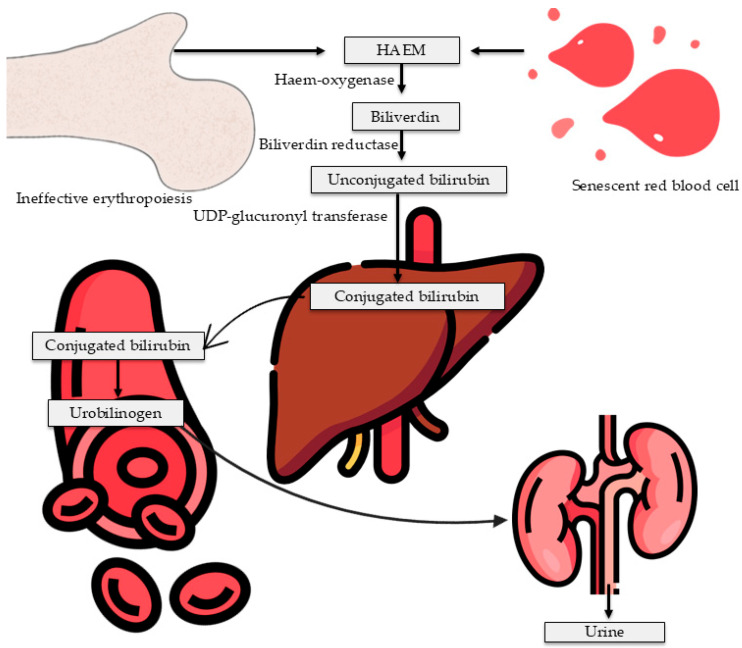
A. Schematic depiction of how ineffective erythropoiesis and aging red blood cells contribute to the elevation of bilirubin. UDP: uridine diphosphate. Figure created using FLATICON and BIOICONS.

Endothelial dysfunction suppresses the release of vasodilators, such as prostacyclin, and promotes the release of vasoconstrictors, like thromboxane [85]. The activity promotes vasoconstriction of hepatic blood vessels, thereby inducing hypoxia, necrosis, and hepatocyte degeneration. This subsequently increases the levels of AST and ALT in the blood, both of which reflect liver injury [17]. The significant increase in ALP found in PE women likely indicates both hepatic impairment and placental involvement, as placental isoforms of ALP are physiologically elevated during pregnancy [86,87]. Therefore, PE-induced liver dysfunction is mediated by endothelial injury and inflammation that impair hepatic function [88,89]. Furthermore, vascular damage in PE impairs normal hepatic blood flow, leading to hepatocyte injury and reduced liver function, including bilirubin processing and clearance. For example, other studies have shown reduced haemoglobin levels in PE compared to controls [86,87], suggesting that haemoglobin may have been broken down through haemolysis, as reflected in elevated blood bilirubin levels. Previous reports have shown a substantial increase in red blood cell breakdown in severe cases, such as HELLP syndrome, resulting in elevated circulating bilirubin levels [90]. Altogether, impaired hepatic clearance and increased haemoglobin breakdown resulted in the accumulation of serum bilirubin in PE [9,17,48,52]. Our results are consistent with previous reports that showed higher bilirubin levels in PE compared with normotensive individuals [50,52,91]. This activity is associated with an increased rate of haemolysis (Figure 9).

Indeed, elevated AST, ALT, ALP, and bilirubin in PE indicate compromised hepatic clearance or increased haemolysis, both of which suggest liver injury and dysfunction.

This study has several strengths, the main one being that it is the first meta-analysis to examine the impact of PE on liver function in pregnant women. Additionally, it has employed an extensive literature review, with a substantial sample size, and strict adherence to the MOOSE guideline. The studies included were of good quality, as none were rated as poor on the Newcastle–Ottawa Scale. By integrating data from multiple countries (Figure 2), it provides a comprehensive overview of PE’s involvement in hepatic health during pregnancy on a global scale. Interestingly 52% and 48% of studies were of higher and moderate quality, respectively. Nevertheless, limitations must also be acknowledged. The evidence presented substantial heterogeneity across all outcomes. However, subgroup analysis and meta-regression were thoroughly conducted to identify sources and their association with the effect size. To some extent, variation in outcomes was attributed to the study design, particularly the cohorts and the gestational age at diagnosis of PE (more than 30 weeks), which were noted as potential sources of heterogeneity for bilirubin. However, for other outcomes (AST, ALT, and ALP), the variation could not be explained through the subgroup. A thorough meta-regression showed an association between moderators and effect sizes across outcomes. We noted publication bias, as evidenced by funnel plot asymmetry, Egger’s regression, Beggs’s test, trim-and-fill, and fail-safe n assessment. Not all studies documented baseline data, such as blood pressure, BMI, maternal age, and gestational age at the time of PE diagnosis, which remain critical for PE diagnosis. The information about the assay method used to assess these liver enzyme tests is limited; therefore, it was not used for subgroup analysis or meta-regression. As evidence is gathered from observational studies, it is essential to interpret the results with caution, as they do not establish causality and are susceptible to confounding, selection bias, and measurement bias. Moreover, for cross-sectional studies, while they may suggest an association, they fail to assess incidence or risk.

## 5. Conclusions

This meta-analysis reveals that PE induces liver dysfunction during pregnancy, as evidenced by significant elevations in liver function tests, including AST, ALT, ALP, and total bilirubin. These findings highlight the importance of monitoring liver function in pregnancy, especially if this is associated with hypertensive disorders. However, substantial variability and publication bias warrant careful interpretation. Based on evidence from observational studies, we recommend that future research focus on high-quality studies, including clinical trials, to investigate the potential treatment of liver dysfunction in PE during pregnancy and to prevent severe maternal and foetal complications.

## Figures and Tables

**Table 1:** PECOS criteria.

Population (P)	Pre-eclamptic pregnant women
Exposure (E)	Pre-eclampsia
Comparison (C)	Healthy pregnant women (normotensive)
Outcome (O)	Liver function (AST, ALT, ALP, and bilirubin)
Study design (S)	Cross-sectional, case–control, and cohort

**Table 2:** Overview of features of included studies (*n* = 45).

Author Name and Publication Year	Country	Study Design	PE Group	Normotensive Group	Age (Years) PE Group	Age (Years) Normotensive Group	SBP and DBP (mmHg) PE Group	SBP and DBP (mmHg) Normotensive Group	Markers
Atiba et al., 2016 [28]	Nigeria	Cross-sectional	98	115	28.87 ± 4.33	28.87 ± 6.62	166.15 ± 9.40 99.80 ± 2.66	117.83 ± 13.03 70.87 ± 9.65	AST
Ekun et al., 2018 [68]	Nigeria	Cross-sectional	49	50	33.18 ± 4.55	32.44 ± 4.99	172.55 ± 24.16 112.47 ± 17.73	113.02 ± 9.95 70.20 ± 9.09	AST and ALT
Asha and Varghese, 2017 [69]	India	Case–control	50	50	NR	NR	NR	NR	AST, ALT, and ALP
Chen et al., 2022 [32]	China	Case–Control	73	73	30.17 ± 4.33	29.87 ± 4.14	116.43 ± 10.3 71.96 ± 9.65	106.07 ± 11.91 65.59 ± 9.78	AST and ALT
Cho et al., 2018 [42]	South Korea	Cohort	3973	192,571	30.92 ± 3.75	30.25 ± 3.38	117.60 ± 13.46 74.76 ± 10.00	109.6 ± 10.69 69.00 ± 7.95	AST and ALT
Hamed et al., 2023 [46]	Libya	Cross-sectional	60	40	33.62 ± 6.50	28.77 ± 7.26	NR	NR	ALT and AST
Fang et al., 2024 [47]	China	Retrospective cohort	53	898	29.23 ± 1.65	29.77 ± 1.77	112.5 ± 2.92 72.5 ± 2.92	108 ± 3.45 67.5 ± 2.84	AST, ALT, and ALP
Albayrak and Arslan, 2025 [71]	Türkiye	Retrospective case–control	207	205	31.2 ± 6.89	29.5 ± 5.13	156.5 ± 9.2 96.8 ± 7.5	110.6 ± 7.3 63.5 ± 6.8	AST, ALP, and ALP
Lu et al., 2025 [70]	China	Retrospective case–control	113	217	31.32 ± 5.10	31.53 ± 4.06	140 ± 0.75 90 ± 0.2	140 ± 0.67 90 ± 0.33	AST, ALT, ALP, and bilirubin
Hassanpour and Karami, 2018 [48]	Iran	Case–control	50	49	NR		NR	NR	ALT, AST, ALP, and bilirubin
Hassen et al., 2022 [29]	Ethiopia	Cross-sectional	51	51	32.9 ± 6.3	29.5 ± 3.3	142.8 ± 6.34 92.8 ± 5.22	NR	ALT, AST, ALP, and bilirubin
Hendawy et al., 2020 [38]	Saudi Arabia	Cross-sectional	100	100	NR	NR	NR	NR	ALT and AST
İpek et al., 2024 [45]	Turkey	Case–Control	92	91	31.0 ± 10.0	27.0 ± 8.0	NR	NR	ALT and AST
Khan et al., 2023 [31]	India	Cross-sectional	150	150	29.24 ± 3.43	29.09 ± 3.08	154.28 ± 22.31 100.21 ± 12.34	119.08 ± 12.20 74.34 ± 6.22	ALT and AST
Mishra et al., 2021 [33]	India	Case–control	33	25	30 ± 5	NR	151.3 ± 3.0 70.83 ± 3.41	100.0 ± 1.82 111.2 ± 4.94	AST and bilirubin
Mondal et al., 2016 [30]	Bangladesh	Cross-sectional	50	50	26.58 ± 3.97	26.06 ± 5.02	156.56 ± 14.05 104.69 ± 8.88	107.5 ± 12.95 69.69 ± 7.82	ALT and bilirubin
Munazza et al., 2013 [52]	Pakistan	Comparative cross-sectional	50	50	15–45	15–45	166.60 ± 24.04 106.50 ± 13.18	116.80 ± 9.022 73.44 ± 7.29	ALT, AST, and bilirubin
Qassim and Ameen, 2021 [34]	Iraq	Case–control	50	50	16–40	16–40	NR	NR	ALT and AST
Sakr et al., 2019 [51]	Egypt	Cross-sectional	25	25	18–35	18–35	NR	NR	AST and ALT
Singh and Rachna, 2021 [72]	India	Case–control	30	30	18 and above	18 and above	167.33 ± 25.45 103.33 ± 12.41	113.33 ± 7.58 75.00 ± 5.08	ALT, AST, and ALP
Sultana et al., 2021 [35]	Bangladesh	Case–control	50	50	24.66 ± 3.22	24.06 ± 3.71	NR	NR	ALT
Uckan and Sahin, 2018 [36]	Turkey	Case–control	30	30	30.7 ± 8.01	28.5 ± 8.03	175.6 ± 16.82 95.8 ± 7.97	122.5 ± 7.2 72.6 ± 8.73	AST and ALT
Udenze et al., 2014 [37]	Nigeria	Case–control	21	21	31.73 ± 5.5	32.57 ± 5.5	153.4 ± 52 99.1 ± 34.5	116.19 ± 12.00 70.7 ± 1.12	AST, ALT, and ALP
Al Ghazali et al., 2014 [39]	Iraq	Cross-sectional	53	21	25.23 ± 6.23	27.28 ± 6.14	168.58 ± 20.54 110.39 ± 9.025	117.4 ± 9.95 76.7 ± 9.13	AST and ALT
Nie et al., 2025 [40]	China	Retrospective cohort	1200	11,499	30.09 ± 5.40	29.19 ± 4.64	134.76 ± 7.95 84.53 ± 7.36	115.28 ± 12.95 71.56 ± 6.57	ALT, AST, and bilirubin
Shahid et al., 2019 [41]	Pakistan	Prospective cohort	63	17	27.05 ± 5.89	30.65 ± 9.40	125.44 ± 16.23 83.95 ± 7.6	108.22 ± 5.89 79.55 ± 8.45	ALP and bilirubin
Taimoor et al., 2017 [50]	Pakistan	Comparative cross-sectional	50	50	25.92 ± 5.56	25.92 ± 5.56	NR	NR	AST, ALP, and bilirubin
Walle et al., 2022 [49]	Ethiopia	Comparative cross-sectional	63	63	28.1 ± 4.61	27.5 ± 4.77	145.4 ± 8.6 94.5 ± 6.1	103.7 ± 10.2 69.5 ± 7.5	AST, ALT, and bilirubin
Haggai et al., 2022 [44]	Israel	Prospective cohort	36	37	31.17 ± 7.60	28.59 ± 4.77	156.0 ± 14.2 94.25 ± 12.1	117.92 ± 10.8 70.0 ± 8.4	AST and ALT
Zhang et al., 2025 [43]	China	Prospective cohort	1588	35,858	32.4 ± 4.2	32.4 ± 4.2	NR	NR	AST and ALT
Zhestkova et al., 2023 [53]	Russia	Cross-sectional	73	50	30.65 ± 2.39	31.75 ± 2.01	150.23 ± 6.73 93.43 ± 3.06	108 ± 3.45 65 ± 2.9	ALP and bilirubin
Singh et al., 2017 [55]	India	Cross-sectional	70	70	25.6 ± 3.7	25.1 ± 3.9	156.5 ± 18.4 102.0 ± 16.3	117.2 ± 8.9 76.7 ± 4.5	AST, ALT, ALP, and bilirubin
Zhang et al., 2022 [54]	China	Cohort	244	5281	31.9 ± 4.5	30.9 ± 4.0	NR	NR	AST, ALT, and ALP
Nainani and Bhargava, 2019 [56]	India	Comparative cross-sectional	39	100	20–45	NR	NR	NR	AST, ALT, ALP and bilirubin
Ohotu et al., 2023 [57]	Nigeria	Cross-sectional	35	35	NR	NR	169.56 ± 20.02 107.45 ± 8.14	117.42 ± 6.01 75.36 ± 8.20	AST, ALT, and ALP
Salman, 2016 [58]	Iraq	Comparative cross-sectional	40	40	NR	NR	158.5 ± 8.92 111.75 ± 7.1	108.75 ± 7.9 73.0 ± 5.16	AST, ALT and bilirubin
Das et al., 2013 [60]	India	Cross-sectional	50	50	NR	NR	164.58 ± 22.04 104.48 ± 12.16	114.72 ± 8.01 72.41 ± 6.28	ALT, AST, ALP, and bilirubin
Roy and Lodhi, 2019 [61]	India	Cross-sectional	30	30	27.53 ± 5.15	29.23 ± 6.08	NR	NR	AST, ALT, and bilirubin
Al-Sultan et al. [62]	Iraq	Comparative cross-sectional	60	35	NR	NR	NR	NR	AST, ALT, and ALP
Afroz et al., 2021 [63]	India	Cross-sectional	50	50	28.0 ± 5.93	26.20 ± 5.32	153.4 ± 16.7 91.7 ± 5.2	113.4 ± 4.5 74.1 ± 6.6	AST and ALT
Saha et al., 2022 [59]	India	Cross-sectional	60	60	32.42 ± 6.45	26.44 ± 8.14	146.32 ± 8.21 94.46 ± 6.84	110.86 ± 12.54 79.44 ± 6.47	AST, ALT, ALP, and bilirubin
Al-Jameil et al., 2015 [64]	Saudi Arabia	Cross-sectional	40	40	31.55 ± 6.14	31.20 ± 5.84	167.0 ± 24.43 98.51 ± 11.16	113.56 ± 13.93 67.66 ± 9.38	AST, ALT, ALP, and bilirubin
Makuyana et al., 2002 [65]	Zimbabwe	Cross-sectional	38	72	27 ± 6	25 ± 6	165 ± 20 109 ± 13	118 ± 11 74 ± 8	AST, ALT, ALP, and bilirubin
Hazari et al., 2014 [66]	India	Cross-sectional	40	40	32.42 ± 6.45	25.13 ± 2.34	146.32 ± 8.21 94.46 ± 6.84	110.0 ± 10.4 67.4 ± 4.8	AST, ALP. ALP and bilirubin
Edebiri et al., 2025 [67]	Nigeria	Comparative cross-sectional	40	20	NR	NR	NR	NR	ALT, AST, and ALP

SBP: Systolic Blood Pressure; DBP: Diastolic Blood Pressure; PE: Pre-eclampsia; NR: Not Reported; ALT: Alanine Aminotransferase; AST: Aspartate Aminotransferase; ALP: Alkaline Phosphatase. Age, SBP, and DBP are reported as means ± SD or range.

**Table 3:** Meta-Regression Examining the Influence of Moderator Variables on the Effect Estimates.

Outcomes	Moderators	*β*	se	*p*	Lower CI	Upper CI
AST	Intercept	2.827	0.335	<0.001	2.170	3.484
	Study design	−0.630 *	0.183	<0.001	−0.989	−0.270
	Intercept	1.7535	0.424	<0.001	0.923	2.584
	Maternal age	0.0198	0.172	0.909	−0.318	0.358
	Intercept	2.306	0.322	<0.001	1.675	2.936
	BMI	−0.281	0.157	0.075	−0.589	0.028
	Intercept	2.078	0.2617	<0.001	1.565	2.591
	Gestation age	−0.120	0.0910	0.187	−0.298	0.058
	Intercept	0.182	0.470	0.698	−0.739	1.103
	Quality	1.084 *	0.299	<0.001	0.498	1.670
	Intercept	2.131	0.566	<0.001	1.021	3.241
	Continent	−0.179	0.293	0.542	−0.753	0.395
ALT	Intercept	3.01	0.573	<0.001	1.884	4.131
	Study design	−1.08 *	0.459	0.019	−1.979	−0.180
	Intercept	1.443	0.451	0.001	0.558	2.327
	Maternal age	0.137	0.203	0.499	−0.260	0.534
	Intercept	2.454	0.318	<0.001	1.831	3.078
	BMI	−0.406 *	0.147	0.006	−0.694	−0.117
	Intercept	1.340	0.363	<0.001	0.628	2.052
	Gestation age	0.228	0.190	0.231	−0.145	0.601
	Intercept	0.653	0.547	0.232	−0.419	1.725
	Quality	0.729 *	0.353	0.039	0.037	1.421
	Intercept	1.8034	0.650	0.006	0.529	3.078
	Continent	−0.0452	0.338	0.894	−0.709	0.618
ALP	Intercept	1.816	0.583	0.002	0.673	2.958
	Study design	−0.231	0.297	0.435	−0.813	0.350
	Intercept	3.027	0.673	<0.001	1.709	4.345
	Maternal age	−0.720 *	0.278	0.010	−1.266	−0.175
	Intercept	1.689	0.539	0.002	0.633	2.745
	BMI	−0.177	0.286	0.535	−0.737	0.383
	Intercept	0.993	0.490	0.043	0.032	1.954
	Gestation age	0.220	0.226	0.330	−0.223	0.663
	Intercept	−0.705	0.753	0.349	−2.181	0.772
	Quality	1.444 *	0.488	0.003	0.488	2.400
	Intercept	−2.14	0.938	0.022	−3.982	−0.305
	Continent	2.07 *	0.528	<0.001	1.032	3.104
Bilirubin	Intercept	1.189	0.234	<0.001	0.730	1.647
	Study design	−0.384 *	0.134	0.004	−0.647	−0.122
	Intercept	1.237	0.294	<0.001	0.660	1.814
	Maternal age	−0.304 *	0.129	0.019	−0.558	−0.050
	Intercept	0.856	0.277	0.002	0.312	1.399
	BMI	−0.142	0.145	0.328	−0.427	0.143
	Intercept	0.5900	0.248	0.017	0.104	1.075
	Gestation age	0.0151	0.135	0.911	−0.250	0.280
	Intercept	−0.607	0.338	0.073	−1.269	0.056
	Quality	0.761 *	0.206	<0.001	0.358	1.164
	Intercept	−0.0808	0.662	0.903	−1.379	1.217
	Continent	0.3815	0.354	0.282	−0.313	1.076

BMI: body mass index; AST: aspartate aminotransferase; ALT: alanine aminotransferase; ALP: alkaline phosphatase; CI: confidence interval; se: standard error; *: shows statistically significant effect.

## Data Availability

All data supporting this manuscript are provided in the Supplementary Materials.

## Supplementary Materials

The following supporting information can be downloaded at: https://www.mdpi.com/article/10.3390/life16020223/s1, Supplementary File S1: MOOSE Checklist; Supplementary File S2: Table S1: Subgroup analysis showing the effect of different factors on liver function, Table S2: Quality assessment of Cohort Studies, Table S3: Quality assessment of Cross-sectional studies, Table S4: Quality assessment of case–control studies.
