# Supplementary material for: Pre-Eclampsia-Induced Maternal Liver Dysfunction: Systematic Review, Meta-Analysis and Meta-Regression of Observation Studies
Source: Life (Basel). 2026 Jan 29;16(2):223. doi: 10.3390/life16020223 (PMC12941613; doi:10.3390/life16020223)
Supplement: Supplementary file 1 [file life-16-00223-s001.zip › Supplementary File S1.pdf]

MOOSE Checklist for Meta-analyses of Observational Studies

Item No	Recommendation	Reported on Page No
Reporting of background should include		
1	Problem definition	4
2	Hypothesis statement	-
3	Description of study outcome(s)	5
4	Type of exposure or intervention used	5
5	Type of study designs used	5
6	Study population	5-6
Reporting of search strategy should include		
7	Qualifications of searchers (eg, librarians and investigators)	Title page
8	Search strategy, including time period included in the synthesis and key words	5-6
9	Effort to include all available studies, including contact with authors	6, Fig 1
10	Databases and registries searched	5
11	Search software used, name and version, including special features used (eg, explosion)	6
12	Use of hand searching (eg, reference lists of obtained articles)	7-8
13	List of citations located and those excluded, including justification	6, Fig 1
14	Method of addressing articles published in languages other than English	-
15	Method of handling abstracts and unpublished studies	-
16	Description of any contact with authors	-
Reporting of methods should include		
17	Description of relevance or appropriateness of studies assembled for assessing the hypothesis to be tested	5-6
18	Rationale for the selection and coding of data (eg, sound clinical principles or convenience)	6-7
19	Documentation of how data were classified and coded (eg, multiple raters, blinding and interrater reliability)	6
20	Assessment of confounding (eg, comparability of cases and controls in studies where appropriate)	6
21	Assessment of study quality, including blinding of quality assessors, stratification or regression on possible predictors of study results	Appendix 4-7
22	Assessment of heterogeneity	Appendix, Table S1, 2-3
23	Description of statistical methods (eg, complete description of fixed or random effects models, justification of whether the chosen models account for predictors of study results, dose-response models, or cumulative meta-analysis) in sufficient detail to be replicated	6-7
24	Provision of appropriate tables and graphics	Figs 1-7 Tables 1-2
Reporting of results should include		
25	Graphic summarizing individual study estimates and overall estimate	Figs 2-6
26	Table giving descriptive information for each study included	Table 2
27	Results of sensitivity testing (eg, subgroup analysis)	20
28	Indication of statistical uncertainty of findings	-

Item No	Recommendation	Reported on Page No
Reporting of discussion should include		
29	Quantitative assessment of bias (eg, publication bias)	17-19, Fig 6
30	Justification for exclusion (eg, exclusion of non-English language citations)	7
31	Assessment of quality of included studies	Appendix, 4-7x
Reporting of conclusions should include		
32	Consideration of alternative explanations for observed results	21-22
33	Generalization of the conclusions (ie, appropriate for the data presented and within the domain of the literature review)	23
34	Guidelines for future research	-
35	Disclosure of funding source	25

From: Stroup DF, Berlin JA, Morton SC, et al, for the Meta-analysis Of Observational Studies in Epidemiology (MOOSE) Group. Meta-analysis of Observational Studies in Epidemiology. A Proposal for Reporting. *JAMA*. 2000;283(15):2008-2012. doi: 10.1001/jama.283.15.2008.
