# Supplementary material for: Pre-Eclampsia-Induced Maternal Liver Dysfunction: Systematic Review, Meta-Analysis and Meta-Regression of Observation Studies
Source: Life (Basel). 2026 Jan 29;16(2):223. doi: 10.3390/life16020223 (PMC12941613; doi:10.3390/life16020223)
Supplement: Supplementary file 1 [file life-16-00223-s001.zip › Supplementary File S2.pdf]

Pre-Eclampsia-Induced Maternal Liver Dysfunction: Systematic Review, Meta-Analysis and Meta-Regression of Observation Studies

Kay-Lee E. Strauss[1], Wendy N. Phoswa[1], Kabelo Mokgalaboni[1*]

[1]Department of Life and Consumer Sciences, College of Agriculture and Environmental Sciences, University of South Africa, Florida Campus, Roodepoort, 1710, South Africa.
68188242@mylife.unisa.ac.za , mokgak@unisa.ac.za , phoswwn@unisa.ac.za

*Correspondence: mokgak@unisa.ac.za, Calabash Building, Office 02-047

Table S1: Subgroup analysis showing effect of different factors on liver function

		Effect Size	Std. Error	Z	*P*	95% Confidence Interval		
						Lower	Upper	I^2
	**Design**							
**AST**	Case control	1.199	0.2666	4.496	<0.001	0.676	1.721	93.8
	Cohort	0.971	0.2994	3.243	0.001	0.384	1.558	99.7
	Cross Sectional	2.462	0.3433	7.171	<0.001	1.789	3.135	99.9
	Overall	1.810	0.1512	11.974	<0.001	1.514	2.107	99.1
	**Quality**							
	High	1.266	0.1910	6.630	<0.001	0.892	1.641	99.4
	Moderate	2.466	0.3683	6.696	<0.001	1.744	3.187	98.0
	Overall	1.810	0.1512	11.974	<0.001	1.514	2.107	99.1
	**Continents**							
	Africa	1.738	0.4398	3.952	<0.001	0.876	2.600	96.8
	Asia	1.960	0.1799	10.898	<0.001	1.608	2.313	99.3
	Europe	0.730	0.3524	2.070	0.038	0.039	1.420	92.2
	Overall	1.810	0.1512	11.974	<0.001	1.514	2.107	99.1
	**Maternal age (years)**							
	< 30 years	1.843	0.3098	5.949	<0.001	1.236	2.450	95.7
	> 30 years	1.836	0.1954	9.396	<0.001	1.453	2.219	99.4
	NR	1.719	0.7420	2.316	0.021	0.264	3.173	97.8
	Overall	1.810	0.1512	11.974	<0.001	1.514	2.107	99.1
	**BMI**							
	Normal	0.926	0.2203	4.202	<0.001	0.494	1.357	97.2
	NR	1.981	0.2486	7.971	0.001	1.494	2.469	98.2
	Obese	1.773	0.9540	1.859	0.063	-0.096	3.643	95.7

	Overweight	2.090	0.3389	6.166	<0.001	1.425	2.754	97.1
	**Gestation duration at diagnosis (weeks)**							
	<20	1.790	0.7594	2.357	0.018	0.302	3.278	97.6
	>20	2.008	0.2385	8.421	<0.001	1.541	2.475	99.3
	>30	1.705	0.7985	2.135	0.033	0.139	3.270	98.0
	NR	1.012	0.2769	3.656	<0.001	0.470	1.555	91.9
	Overall	1.810	0.1512	11.974	<0.001	1.514	2.107	99.1
ALT	Case control	1.384	0.3427	4.038	<0.001	0.712	2.055	96,4
	Cohort	0.851	0.3946	2.156	0.031	0.077	1.624	99.9
	Cross Sectional	2.256	0.3210	7.027	<0.001	1.627	2.885	97.9
	Overall	1.729	0.1739	9.944	<0.001	1.388	2.070	99.3
	< 30 years	1.607	0.2759	5.826	<0.001	1.067	2.148	95.9
	> 30 years	1.887	0.2764	6.827	<0.001	1.345	2.429	99.7
	NR	1.731	0.4808	3.601	<0.001	0.789	2.674	97.2
	Overall	1.729	0.1739	9.944	<0.001	1.388	2.070	99.3
	Africa	1.359	0.5069	2.681	0.007	0.365	2.352	97.9
	Asia	2.030	0.2063	9.840	<0.001	1.626	2.434	99.5
	Europe	0.096	0.4025	0.240	0.811	-0.692	0.885	94.4
	Overall	1.729	0.1739	9.944	<0.001	1.388	2.070	99.3
	<20	5.700	2.6694	2.135	0.033	0.468	10.931	97.2
	>20	1.779	0.2235	7.961	<0.001	1.341	2.217	99.5
	>30	0.949	0.4439	2.137	0.033	0.079	1.819	95.8
	NR	1.561	0.4495	3.472	<0.001	0.680	2.442	97.9
	Overall	1.729	0.1739	9.944	<.0001	1.388	2.070	99.3
	High	1.381	0.2345	5.889	<0.001	0.921	1.841	99.6

	Moderate	2.183	0.3285	6.646	<0.001	1.539	2.827	97.7
	Overall	1.729	0.1739	9.944	<0.001	1.388	2.070	99.3
	Normal	0.667	0.3350	1.991	0.046	0.010	1.324	99.7
	NR	2.092	0.3101	6.747	<0.001	1.484	2.700	98.1
	Obese	1.892	1.9729	0.959	0.337	-1.974	5.759	98.7
	Overweight	1.846	0.3610	5.113	<0.001	1.138	2.553	97.4
	Overall	1.729	0.1739	9.944	<0.001	1.388	2.070	99.3
Bilirubin	Case control	0.623	0.2421	2.574	0.010	0.149	1.098	84.0
	Cohort	-0.169	0.0291	-5.805	<0.001	-0.226	-0.0112	0.0
	Cross Sectional	0.831	0.1769	4.698	<0.001	0.484	1.178	89.8
	Overall	0.621	0.1337	4.644	<0.001	0.359	0.883	93.7
	< 30 years	1.080	0.3104	3.479	<0.001	0.472	1.688	94.6
	> 30 years	0.322	0.1330	2.421	0.015	0.061	0.582	88.8
	NR	0.636	0.2543	2.502	0.012	0.138	1.135	73.4
	Overall	0.621	0.1337	4.644	<0.001	0.359	0.883	93.7
	Africa	0.306	0.2403	1.271	0.204	-0.165	0.776	80.6
	Asia	0.695	0.1538	4.518	<0.001	0.393	0.996	94.5
	Overall	0.621	0.1337	4.644	<0.001	0.359	0.883	93.7
	<20	0.519	0.4536	1.145	0.252	-0.370	1.408	84.7
	>20	0.583	0.1602	3.638	<0.001	0.269	0.897	94.2
	>30	0.772	0.1553	4.970	<0.001	0.467	1.076	51.1
	NR	0.778	0.9225	0.843	0.399	-1.030	2.586	96.3
	Overall	0.621	0.1337	4.644	<0.001	0.359	0.883	93.7
	High	0.146	0.1124	1.301	0.193	-0.074	0.367	82.6
	Moderate	1.006	0.1941	5.185	<0.001	0.626	1.387	90.3
	Overall	0.621	0.1337	4.644	<0.001	0.359	0.883	93.7
	Normal	0.674	0.2650	2.542	0.011	0.154	1.193	73.2
	NR	0.900	0.2291	3.929	<0.001	0.451	1.349	93.0

	Obese	0.438	0.2263	1.936	0.053	-0.005	0.882	.
	Overweight	0.280	0.1664	1.682	0.093	-0.046	0.606	89.9
	Overall	0.621	0.1337	4.644	<0.001	0.359	0.883	93.7
	**Design**							
ALP	Case control	1.689	0.4937	3.420	<0.001	0.721	2.656	96.3
	Cohort	0.972	0.4295	2.263	0.024	0.130	1.814	99.3
	Cross Sectional	1.413	0.4781	2.956	0.003	0.476	2.350	97.3
	Overall	1.423	0.2326	6.118	<0.001	0.967	1.879	98.3
	**Continent**							
	Africa	-1.646	1.2677	-1.299	0.194	-4.131	0.838	98.2
	Asia	1.800	0.2422	7.432	<0.001	1.325	2.275	98.4
	Overall	1.423	0.2326	6.118	<0.001	0.967	1.879	98.3
	**Maternal age**							
	<20 years	2.308	0.6125	3.769	<0.001	1.108	3.509	99.5
	>20 years	1.279	0.3725	3.433	<0.001	0.549	2.009	97.8
	>30 years	1.303	0.3849	3.385	<0.001	0.548	2.057	87.7
	NR	0.549	0.8959	0.613	0.540	-1.207	2.305	96.3
	Overall	1.423	0.2326	6.118	<.001	0.967	1.879	98.3
	**Quality**							
	High	.751	.03296	2.277	0.023	0.105	1.397	99.0
	Moderate	2.208	0.3722	5.933	<0.001	1.479	2.938	95.8
	Overall	1.423	0.2326	6.118	<0.001	0.967	1.879	98.3
	**BMI**							
	Normal	1.114	0.8366	1.331	0.183	-0.526	2.753	99.8
	NR	1.550	0.3567	4.344	<0.001	0.850	2.249	96.9
	Obese	1.042	0.2383	4.374	<0.001	0.575	1.509	.

	Overweight	1.241	0.4329	2.868	0.004	0.393	2.090	96.3
	Overall	1.423	0.2326	6.118	<0.001	0.967	1.879	98.3

Table S2: Quality assessment of Cohort Studies

	**Selection**				**Comparability**	**Outcome**			**Score**
	Representativeness of exposed cohort (1)	Selection of non-exposed cohort (1)	Ascertainment of exposure (1)	Demonstration that outcome of interest was not present at the start of study (1)	Comparability of cohorts on the basis of the design or analysis controlled for confounders (2)	Assessment of outcome (2)	Was follow-up long enough for outcomes to occur (1)	Adequacy of follow-up cohorts (1)	10
Cho et al.. 2022	★	★	★	★	★★	★★	★	★	10/10
Fang et al.. 2024	★	★	★	★	★★	★★	★	★	10/10
Haggai et al.. 2022	★	★	★	★	★★	★★	★	★	10/10
Nie et al.. 2025	★	★	★	★	★★	★★	★	★	10/10
Zhang et al.. 2022	★	★	★	★	★★	★★	★	★	10/10
Shahid et al.. 2019	★	★	★	★	★	★★	★	☆	8/10
Zhang et al.. 2025	★	★	★	★	★★	★★	★	★	10/10

Table S3: Quality assessment of Cross-sectional studies

	**Selection**				**Comparability**	**Outcome**			**Score**
	Representativeness of the sample (1)	Sample size (1)	Non-included subjects (1)	Ascertainment of exposure (1)	Based on design and analysis (2)	Assessment of outcome (2)	Statistical test (1)	Reporting outcomes and precision (1)	10
Atiba et al.. 2016	★	☆	☆	★	★	★★	★	★	7/10
Hamed et al.. 2023	★	☆	☆	★	★	★★	★	★	7/10
Hassen et al.. 2022	★	★	☆	★	★★	★★	★	★	9/10
Makuyana et al.. 2002	★	★	☆	★	★	★★	★	★	8/10
Saha. 2022	★	★	☆	★	★	★★	★	★	8/10
Salman. 2016	★	☆	☆	★	★	★★	★	★	7/10
Hazari et al.. 2014	★	☆	☆	★	★	★★	★	★	7/10
Afroz et al.. 2020	★	☆	☆	★	★	★★	★	★	7/10
Al-Sultan et al.. 2025	★	☆	☆	★	★	★★	★	★	7/10
Nainani and Bhargava. 2019	★	☆	☆	★	★	★★	★	★	7/10
Edebiri et al.. 2025	★	★	☆	★	★	★★	★	★	8/10
Singh et al.. 2017	★	☆	☆	★	★	★★	★	★	7/10
Al-Jameil et al.. 2015	★	☆	☆	★	★	★★	★	★	7/10

Khan et al.. 2023	★	☆	☆	★	★	★★	★	★	7/10
Roy and Lodhi. 2019	★	☆	☆	★	★	★★	★	★	7/10
Mondal et al.. 2016	★	☆	☆	★	★	★★	★	★	7/10
Munazza et al.. 2013	★	☆	☆	★	★	★★	★	★	7/10
Taimoor et al.. 2017	★	☆	☆	★	★	★★	★	★	7/10
Walle et al.. 2022	★	☆	☆	★	★	★★	★	★	7/10
Sakr et al.. 2019	★	☆	☆	★	★	★★	★	★	7/10
Zhestkova et al.. 2023	★	☆	☆	★	★	★★	★	★	7/10
Ohotu et al.. 2023	★	★	☆	★	★	★★	★	★	8/10
Hendawy et al.. 2020	★	☆	☆	★	★	★★	★	★	7/10
Das et al.. 2013	★	☆	☆	★	★	★★	★	★	7/10
Ekun et al.. 2018	☆	☆	☆	★	★	★★	★	☆	5/10
Ghazali et al.. 2014	★	☆	☆	★	★	★★	★	★	7/10

Table S4: Quality assessment of case-control studies

	**Selection**				**Comparability**	**Exposure**			**Score**
	Is the case definition adequate?	Representativeness of the case	Selection of controls	Definition of controls	Comparability of cases and controls on the bases of the design or analysis	Ascertainment of exposure	Same method of ascertainment for cases and controls	Non-Response rate	10
Hassanpour and Karami. 2018	★	★	★	★	☆	★★	★	★	8/10
Mishra et al.. 2021	★	★	★	★	★	★★	★	★	9/10
Qassim and Ameen. 2021	★	★	★	★	★	★★	★	★	9/10

Asha and Varghese. 2017	★	☆	★	★	☆	★	★	★	6/10
Singh and Rachna. 2025	★	★	★	★	☆	★★	★	★	8/10
Sultana et al.. 2021	★	★	★	★	★	★★	★	★	9/10
Uckan and Sahin. 2018	★	★	★	★	★	★★	★	★	9/10
Udenze et al.. 2014	★	★	★	★	★	★★	★	★	9/10
Ipek et al.. 2024	★	★	★	★	★	★★	★	★	9/10
Chen et al.. 2022	★	★	★	★	★★	★★	★	★	9/10
Lu et al.. 2025	★	★	★	★	★	★★	★★	★	10/10
Albayrak and Arslan. 2025	★	★	★	★	★★	★	★	★	9/10

References

1. Atiba, A.S.; Abbiyesuku, F.M.; Oparinde, D.P.; 'Niran-Atiba, T.A.; Akindele, R.A. Plasma Malondialdehyde (MDA): An Indication of Liver Damage in Women with Pre-Eclamsia. *Ethiop. J. Health Sci.* **2016**, *26*, 479–486. https://doi.org/10.4314/ejhs.v26i5.10.
2. Hassen, F.S.; Malik, T.; Dejenie, T.A. Evaluation of Serum Uric Acid and Liver Function Tests among Pregnant Women with and without Preeclampsia at the University of Gondar Comprehensive Specialized Hospital, Northwest Ethiopia. *PLoS ONE* **2022**, *17*, e0272165. https://doi.org/10.1371/journal.pone.0272165.
3. Mondal, B.R.; Ahmed, S.; Saha, S.; Parveen, S.I.; Sultana, T.; Rahman, M.Q.; Sarker, U.K.; Aminotransferase, A.A.N.A.; Bilirubin, T. Concentration in Preeclampsia and Eclampsia. *Mymensingh Med. J.* **2016**, *25*, 85–90.
4. Khan, J.A.; Ashraf, A.; Fayaz, F.; Qureshi, W.; Sheikh, A.T. Liver and Renal Biochemical Parameters in Preeclampsia: A Cross Sectional Study. *Int. J. Res. Med. Sci.* **2023**, *11*, 929–935. https://doi.org/10.18203/2320-6012.ijrms20230575.
5. Chen, L.; Pi, Y.; Chang, K.; Luo, S.; Peng, Z.; Chen, M.; Yu, L. Screening Models Combining Maternal Characteristics and Multiple Markers for the Early Prediction of Preeclampsia in Pregnancy: A Nested Case–Control Study. *J. Obstet. Gynaecol.* **2022**, *42*, 1889–1896. https://doi.org/10.1080/01443615.2022.2054675.
6. Mishra, J.; Srivastava, S.K.; Pandey, K.B. Compromised Renal and Hepatic Functions and Unsteady Cellular Redox State during Preeclampsia and Gestational Diabetes Mellitus. *Arch. Med. Res.* **2021**, *52*, 635–640. https://doi.org/10.1016/j.arcmed.2021.03.003.
7. Qassim, A.A.; Ameen, M.A. Evaluation of the Effect of Preeclampsia on Liver and Renal Function Biomarkers Level. *Biochem. Cell. Arch.* **2021**, *21*, 4887–4891.
8. Sultana, R.; Ahmed, S.; Sultana, N.; Diba, F. ALT in Preeclampsia. *Delta Med. Col. J.* **2021**, *9*, 65–68.
9. Uckan, K.; Sahin, H.G. Serum Amyloid A, Procalcitonin, Highly Sensitive C Reactive Protein and Tumor Necrosis Factor Alpha Levels and Acute Inflammatory Response in Patients with Hemolysis, Elevated Liver Enzymes, Low Platelet Count (HELLP) and Eclampsia. *J. Obstet. Gynaecol. Res.* **2018**, *44*, 440–447. https://doi.org/10.1111/jog.13532.
10. Udenze, I.; Arikawe, A.; Azinge, E.; Egbuagha, E. Liver Function Tests in Nigerian Women with Severe Preeclampsia. *J. Clin. Sci.* **2014**, *11*, 7. https://doi.org/10.4103/1595-9587.137241.
11. Hendawy, M.O.; Hussein, S.; Harahsheh, E.A. Relationship between Pre-Eclampsia, Renal Impairment and Hepatic Insufficiency among Pregnant Women in Al-Jouf Area. *J. Pharm. Nutr. Sci.* **2020**, *10*, 295–301.
12. Al Ghazali, B.; Al-Taie, A.A.-H.; Hameed, R.J. Study of the Clinical Significance of Serum Albumin Level in Preeclampsia and in the Detection of Its Severity. *Am. J. Biomed.* **2014**, *2*, 964–974.
13. Nie, L.; Zhang, Z.; Yao, Q.; Chen, H.; Xu, C.; Chen, L.; Liu, C.; Tu, L.; Yi, Y.; Huang, T.; et al. The New Era of Risk Assessment for Hypertension in Pregnancy: From Clinical to Biochemical Markers in a Comprehensive Predictive Model. *Taiwan J. Obstet. Gynecol.* **2025**, *64*, 253–264. https://doi.org/10.1016/j.tjog.2024.10.014.
14. Shahid, S.; Khalid, E.; Fatima, S.S.; Khan, G.M. Evaluation of Soluble TNF-like Weak Inducer of Apoptosis (STWEAK) Levels to Predict Preeclampsia in Early Weeks of Pregnancy. *Eur. J. Obstet. Gynecol. Reprod. Biol.* **2019**, *234*, 165–170. https://doi.org/10.1016/j.ejogrb.2019.01.020.
15. Cho, G.J.; Kim, H.Y.; Park, J.H.; Ahn, K.H.; Hong, S.C.; Oh, M.J.; Kim, H.J. Prepregnancy Liver Enzyme Levels and Risk of Preeclampsia in a Subsequent Pregnancy: A Population-Based Cohort Study. *Liver Int.* **2018**, *38*, 949–954. https://doi.org/10.1111/liv.13617.
16. Zhang, L.; Gao, S.; Luan, Y.; Su, S.; Zhang, E.; Liu, J.; Xie, S.; Zhang, Y.; Yue, W.; Liu, R.; et al. Predictivity of Hepatic Steatosis Index for Gestational Hypertension and Preeclampsia: A Prospective Cohort Study. *Int. J. Med. Sci.* **2025**, *22*, 834–844. https://doi.org/10.7150/ijms.104943.
17. Haggai, C.M.; Inshirah, S.; Jacob, B.; Marwan, O.; Lior, L.; Maya, F.W. Liver Stiffness and Steatosis in Preeclampsia as Shown by Transient Elastography–a Prospective Cohort Study. *Am. J. Obstet. Gynecol.* **2022**, *227*, 515.e1–515.e9. https://doi.org/10.1016/j.ajog.2022.04.048.
18. İpek, G.; Tanaçan, A.; Ağaoğlu, Z.; Gülçin Baştemur, A.; Gülen Yıldız, E.; Şahin, D. The Role of Aspartate Aminotransferase to Platelet Ratio Index (APRI) in the First Trimester for the Prediction of Superimposed

Preeclampsia: A Case-Control Study from a Tertiary Center. *Pregnancy Hypertens.* **2024**, *37*, 101132. https://doi.org/10.1016/j.preghy.2024.101132.

19. Hamed, S.; Hamed, S.S.M.; Khalifa, T.; Ali, M.S. Preeclampsia Symptoms and Liver Function Tests in Women with Pre-Eclampsia: Comparison with a Normal Pregnant Woman. *Sci. J. Fac. Sci.-Sirte Univ.* **2023**, *3*, 141–148. https://doi.org/10.37375/sjfssu.v3i2.101.
20. Fang, Y.; Liu, H.; Li, Y.; Cheng, J.; Wang, X.; Shen, B.; Wang, Q.; Chen, H. A Prediction Model of Preeclampsia in Hyperglycemia Pregnancy. *Diabetes Metab. Syndr. Obes.* **2024**, *17*, 1321–1333. https://doi.org/10.2147/DMSO.S453204.
21. Hassanpour, S.H.; Zeinab Karami, S. Evaluation of Hepatic Biomarkers in Pregnant Women with Preeclampsia. *Gynecol. Obstet.* **2018**, *8*, 1000487. https://doi.org/10.4172/2161-0932.1000487.
22. Walle, M.; Getu, F.; Gelaw, Y.; Getaneh, Z. The Diagnostic Value of Hepatic and Renal Biochemical Tests for the Detection of Preeclampsia Among Pregnant Women Attending the Antenatal Care Clinic at the University of Gondar Comprehensive Specialized Hospital, Gondar, Northwest Ethiopia. *Int. J. Gen. Med.* **2022**, *15*, 7761–7771. https://doi.org/10.2147/IJGM.S382631.
23. Taimoor, A.; Nazir, A.; Raza, N.; Qureshi, S.A.; Ayub, M.; Shirwany, T.A.K. Liver function tests in second and third Trimester Primigravida in normal Pregnancy and Preeclampsia. *Pak. J. Physiol.* **2017**, *13*, 25–28.
24. Sakr, I.H.; Khowailed, A.A.; Kamel, M.M.; Farghaly, E.M.; Farid, Z.E. Endothelial-Platelet Dysfunction as an Indicator of Pre-Eclampsia and Its Severity. *Med. J. Cairo Univ.* **2019**, *87*, 1775–1782. https://doi.org/10.21608/mjcu.2019.53964.
25. Munazza, B.; Raza, N.; Naureen, A.; Khan, S.A.; Fatima, F.; Ayub, M.; Sulaman, M. Liver Function Tests in Preeclampsia. *J. Ayub Med. Coll. Abbottabad* **2013**, *23*, 3–5.
26. Zhestkova, N.V.; Ailamazyan, E.K.; Kuzminykh, T.U.; Marchenko, N.V. Characteristics of Liver Function in Patients with Preeclampsia. *J. Obstet. Women's Dis.* **2023**, *72*, 59–69. https://doi.org/10.17816/JOWD409413.
27. Zhang, Y.; Sheng, C.; Wang, D.; Chen, X.; Chen, X.; Jiang, Y.; Dou, Y.; Wang, Y.; Li, M.; Chen, H.; et al. High-Normal Liver Enzyme Levels in Early Pregnancy Predispose the Risk of Gestational Hypertension and Preeclampsia: A Prospective Cohort Study. *Front. Cardiovasc. Med.* **2022**, *9*, 963957.
28. Singh, A.; Singh, N.P.; Sant, S.K.; Jaiswal, K. Comparative Evaluation of Liver Functions in Pre-Eclamptic and Normal Pregnancy. *J. Evid. Based Med. Healthc.* **2017**, *4*, 5192–5195. https://doi.org/10.18410/jebmh/2017/1037.
29. Nainani, M.; Bhargava, A.K. A Comparison of Liver Enzymes, Bilirubin and Uric Acid in Preeclampsia, Eclampsia and Normotensive Subjects. *Int. J. Clin. Obstet. Gynaecol.* **2019**, *3*, 19–20. https://doi.org/10.33545/gynae.2019.v3.i2a.06.
30. Obiorah Ohotu, E.; Nneoma Micheal, Q.; Sunday Onah, E.; Ogbonna Ogbuabor, A. Comparative Evaluation of Some Liver Enzymes in Preeclamptic and Non-Preeclamptic Patients in the Enugu Metropolis South East Nigeria. *Int. J. Med. Sci. Dent. Res.* **2023**, *6*, 1–7.
31. Ibrahim Salman, M. Evaluation of Liver Function Tests in Normotensive and Hypertensive Pregnancy. *J. Univ. Anbar Pure Sci.* **2016**, *10*, 7–10. https://doi.org/10.37652/juaps.2016.132436.
32. Saha, A.; Gupta, A. Das Study of Changes in Biochemical Parameters of Preeclampsia Patients, a Prospective Five Year Study. *Int. J. Reprod. Contracept. Obstet. Gynecol.* **2022**, *11*, 517. https://doi.org/10.18203/2320-1770.ijrcog20220181.
33. Das, S.; Char, D.; Sarkar, S.; Kanti Saha, T.; Biswas, S.; Rudra, B. Evaluation of Liver Function Test in Normal Pregnancy and Pre-Eclampsia: A Case Control. *IOSR J. Dent. Med. Sci.* **2013**, *12*, 30–32.
34. Roy, N.; Ahsan, L.R. Evaluation of Liver Function Test and Renal Function Test in Pre-Eclampsia: A Case Control Study. *People's J. Sci. Res.* **2019**, *12*, 18–23.
35. Al-Sultan, A.M.; Jankeer, M.H. Evaluation of Liver and Renal Functions Tests in Pregnant Women with Preeclampsia. *Texila Int. J. Public Health* **2025**, *13*. https://doi.org/10.21522/TIJPH.2013.13.01.Art057.
36. Afroz, F.; Sultana, N.; Rahman, A.; Zerin, N.; Mohammad Samsuzzaman, S.; Chowdhury, P.P.; Andalib, M.H.; Morshed, M.; Rahman, M.M.; Kamal, M.M. A Comparative Study of Hepatic Enzymes Between Preeclampsia and Normal Pregnant Women. *J. Dhaka Med. Coll.* **2021**, *29*, 18–22. https://doi.org/10.3329/jdmc.v29i1.51165.

37. Al-Jameil, N.; Tabassum, H.; Al-Mayouf, H.; Al-Otay, L.; Aziz Khan, F. Liver Function Tests as Probable Markers of Preeclampsia—A Prospective Study Conducted in Riyadh. *J. Clin. Anal. Med.* **2015**, *6*, 461–464. https://doi.org/10.4328/JCAM.2200.
38. Makuyana, D.; Mahomed, K.; Shukusho, F.D.; Majoko, F. Liver and Kidney Function Tests in Normal and Pre-Eclamptic Gestation-a Comparison with Non-Gestational Reference Values. *Cent. Afr. J. Med.* **2002**, *48*, 55–59.
39. Hazari, N.R.; Hatolkar, V.S.; Munde, S.M. Study of Serum Hepatic Enzymes in Preeclampsia. *Int. J. Curr. Med. Appl. Sci.* **2014**, *2*, 1–8.
40. Edebiri, O.E.; Adewole, A.S.; Akpe, C.I.; Ehigiamusoe, E.A.; Ikuenobe, V.E.; Ohiwerei, W.O.; Orunta, E.D. Evaluation Of Liver Enzymes (ALP, ALT, AST and GGT) in Preeclamptic Pregnant Women in the Third Trimester Of Pregnancy. *Int. J. Med. Health* **2025**, *4*, 101–113. https://doi.org/10.55606/ijmh.v4i1.5618.
41. Ekun, O.A.; Olawumi, O.M.; Makwe, C.C.; Ogidi, N.O. Biochemical Assessment of Renal and Liver Function among Preeclamptics in Lagos Metropolis. *Int. J. Reprod. Med.* **2018**, *2018*, 1–6. https://doi.org/10.1155/2018/1594182.
42. Asha, N.S.; Varghese, A. Study of Liver Enzymes in Preeclampsia. *J. Med. Sci. Clin. Res.* **2017**, *05*, 15169–15172. https://doi.org/10.18535/jmscr/v5i1.06.
43. Lu, Y.; Yang, L.; Li, X.; Kuai, D.; Tian, W.; Zhang, H. A Prediction Model of Superimposed Preeclampsia in Women with Chronic Hypertension. *Front. Cardiovasc. Med.* **2025**, *12*, 1641662. https://doi.org/10.3389/fcvm.2025.1641662.
44. Albayrak, M.; Arslan, H.F. Useful Biomarkers for Preeclampsia: Evaluating the Diagnostic Potential of FIB-4 and FIB-5 Indices. *Diagnostics* **2025**, *15*, 693. https://doi.org/10.3390/diagnostics15060693.
